# Real-World Evidence of the Efficacy and Safety of Second-Line Therapy After Gemcitabine and Nab-Paclitaxel for Patients with Metastatic Pancreatic Cancer

**DOI:** 10.3390/cancers17172821

**Published:** 2025-08-28

**Authors:** Agata Adamczuk-Nurzyńska, Paweł Nurzyński, Melania Brzozowska, Maciej Jewczak, Andrzej Śliwczyński

**Affiliations:** 1 Department of Oncology, National Medical Institute of the Ministry of the Interior and Administration, 02-507 Warsaw, Poland; pawel.nurzynski@pimmswia.gov.pl; 2 National Medical Institute of the Ministry of the Interior and Administration, 02-507 Warsaw, Poland; mbrzo5@tlen.pl (M.B.); andrzej.sliwczynski@pimmswia.gov.pl (A.Ś.); 3 Department of Spatial Econometrics, Faculty of Economics and Sociology, University of Lodz, 90-255 Lodz, Poland; maciej.jewczak@eksoc.uni.lodz.pl

**Keywords:** pancreatic cancer, second-line therapy, chemotherapy, NALIRI, survival analysis, response to therapy

## Abstract

**Background:** Metastatic pancreatic cancer (mPC) is an aggressive disease with high morbidity and mortality, and long-term survival rates remain poor. New therapeutic options that demonstrate statistically significant improvements in overall survival (OS) and progression-free survival (PFS) are still being sought. Although many first-line (FL) treatment studies exist in the literature, there are almost no prospective studies on second-line (SL) therapy. **Methods:** The aim of this clinical study was to retrospectively analyze the medical history of 251 patients diagnosed with mPC, treated first-line (FL) with GEM-NAB between February 2017 and January 2025. After disease progression, 109 patients received SL treatment. We also present a multivariate analysis based on routinely collected data (demographic, clinical, and laboratory parameters) evaluating their impact on OS and PFS. **Results:** The median age was 66 years (range 37–84 years). The median PFS was 2.33 months (95% CI 1.69–2.97). Specifically, the mPFS was 4.1 months (95% CI 1.31–6.90) for FOLFIRINOX; 2.8 months (95% CI 2.30–3.30) for FOLFIRI; 2.37 months (95% CI 1.66–3.08) for NALIRI; 1.47 months (95% CI 1.18–1.75) for FOLFOX 6; and 0.93 months (95% CI 0.00–2.64) for GEM-cisplatin. The median OS was 5.03 months (95% CI 3.75–6.31). Seven patients achieved a partial response (overall response rate 6%). The most frequent adverse events (AEs) included anemia, fatigue, peripheral neuropathy, neutropenia, and thrombocytopenia. **Conclusions:** As a result, SL treatments were compared, and some statistically significant difference was found between them in PFS time for chemotherapy FOLFIRINOX and GEM + cisplatin. The most frequent AEs occurred during treatment with FOLFIRINOX chemotherapy.

## Simple Summary

The aim of this clinical study was a retrospective analysis of the medical history of 109 patients with metastatic pancreatic cancer (mPC). These patients received second-line (SL) treatment following progression from gemcitabine to nab-paclitaxel (GEM-NAB) as part of the “Treatment of patients with pancreatic adenocarcinoma” drug program initiated by the Minister of Health (MH) between February 2017 and January 2025. We also present a multivariate analysis based on routinely collected data (demographic, clinical, and laboratory parameters) evaluating their impact on overall survival (OS) and progression-free survival (PFS). A statistically significant difference in PFS time was found between the FOLFIRINOX and GEM + cisplatin treatment. The most frequent adverse events (AEs) during FOLFIRINOX chemotherapy treatment were anemia, fatigue, neutropenia, peripheral neuropathy, and thrombocytopenia.

## 1. Introduction

Pancreatic cancer (PC) is a highly aggressive cancer that is characterized by extensive local invasion, early regional, and rapid systemic spread. PC ranks as the seventh most common cause of cancer-related deaths globally. The 5-year survival rate in PC is around 7–8% [1,2]. In Poland, the National Cancer Registry (KRN) reported 3589 cases of PC in 2020. The diagnosis was more common in women, with 1825 cases compared to 1764 in men [3]. Guidelines such as those from the National Comprehensive Cancer Network (NCCN) describe various treatment methods, including surgery, systemic therapies, radiotherapy, and targeted therapy. Complete surgical resection is the only chance for a cure. Unfortunately, a significant number of patients are diagnosed at a stage where they are not eligible for surgical intervention [1,2]. More than 50% of new cases of PC are diagnosed at an advanced stage (locally advanced or metastatic) each year due to a lack of early warning signs and rapid metastasis [4]. PC diagnosed at the metastatic stage is associated with a particularly poor prognosis [5] and an overall survival rate of 6% at 5 years [2,6].

Chemotherapy is usually the only appropriate treatment for metastatic disease. Since 1997, gemcitabine monotherapy (GEM) has represented the standard of care for patients with locally advanced and metastatic pancreatic cancer (mPC) [7].

New therapeutic options for first-line (FL) palliative treatment that demonstrate statistically significant improvements in overall survival (OS) and progression-free survival (PFS) are still being sought. The addition of nanoparticle albumin-bound paclitaxel to gemcitabine was observed to improve patient survival [8]. The phase III MPACT trial, published in 2013, demonstrated that a combination treatment of nab-paclitaxel with gemcitabine (GEM-NAB) achieved better OS compared to GEM; median OS (mOS) was 8.5 vs. 6.7 months. Similarly, the median PFS (mPFS) amounted to 5.5 months vs. 3.7 months [9,10]. The FOLFIRINOX regimen (consisting of 5-FU/leucovorin, oxaliplatin, and irinotecan) was shown to provide better survival rates when compared to GEM [11,12]. In the PRODIGE 4 trial, FOLFIRINOX achieved better OS compared to GEM, with mOS of 11.1 vs. 6.8 months and mPFS of 6.4 vs. 3.3 months [13]. In other words, fit and younger patients with an Eastern Cooperative Oncology Group performance status (ECOG) of 0 to 1 [14,15] are generally treated with triplet therapy, while older, less robust patients are recommended to receive gemcitabine doublet therapy.

As a second-line (SL) treatment, the choice of regimen depends on the FL treatment. SL treatment, including combinations of folic acid, 5-FU, and oxaliplatin (FOLFOX) or nanoliposomal irinotecan (NALIRI) and fluoropyrimidines (if not previously administered), demonstrated acceptable tolerability and modest survival and clinical benefits [16,17,18,19]. Various studies have reported median OS from the start of the SL regimen to death as ranging from 4.8 to 8.8 months [20,21,22]. The NAPOLI-1 trial, a phase 3 international randomized clinical trial (RCT), evaluated the efficacy of nanoliposomal irinotecan combined with 5-FU/LV (NALIRI) as an SL therapy for mPC in patients previously treated with GEM-based regimens. This combination achieved better OS compared to 5-FU/LV, with an mOS of 6.1 vs. 4.2 months [19]. While the literature is rich with studies on FL therapies, prospective investigations into SL therapy remain scarce. The SL chemotherapy decision should be made on a patient basis, taking into account personal preferences, previous chemotherapy toxicity, and goals.

The aim of our retrospective analysis of patients with mPC was to assess the effectiveness and treatment tolerance of SL therapy in the real world. We also present a multivariate analysis based on routinely collected data (demographic, clinical, and laboratory parameters) with an impact on OS and PFS.

## 2. Materials and Methods

### 2.1. Patients and Data Collection

**Figure 1:**
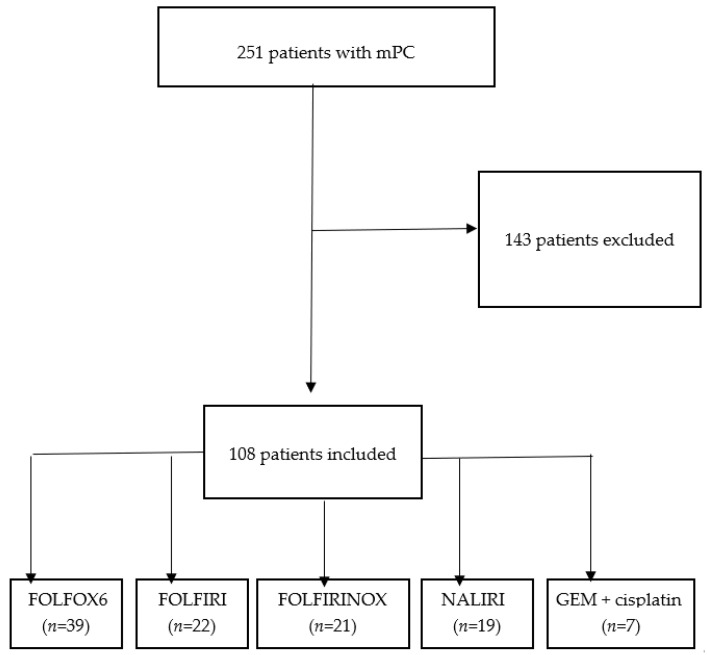
Summary of study design with inclusion criteria.

The aim of our study was a retrospective analysis of the medical history of 251 patients with the diagnosis of mPC who were treated in the FL with GEM-NAB as part of the drug program initiated by the Minister of Health (MH) titled “Treatment of patients with pancreatic adenocarcinoma” between February 2017 and January 2025 in the Oncology Department of the National Medical Institute of the Ministry of the Interior and Administration (PIM MSWiA) in Warsaw. After disease progression, 108 patients received SL treatment (Figure 1).

Inclusion criteria considered: cytologically or histologically confirmed metastatic adenocarcinoma PC, progression after FL therapy GEM-NAB, age ≥ 18 years, and measurable tumor lesions. Cancer staging was determined using computed tomography (CT) scans of the chest, abdomen, and pelvis. Before each subsequent drug administration, patients’ creatinine, bilirubin, ALT, AST, and peripheral blood count were checked.

The treatment was terminated in cases of disease progression, the onset of unacceptable toxicity, or at the patient’s request.

The analyzed medical data encompassed sex, age, ECOG, Body Mass Index (BMI), pathological variables (tumor site, tumor size, tumor stage), sites of metastases, treatment data (type of operation, adjuvant chemotherapy, response to FL therapy), laboratory results, neutrophil to lymphocyte ratio (NLR), calculated as the absolute neutrophil count divided by the absolute lymphocyte count measured in × 10^3^/mL, carbohydrate antigen 19.9 (Ca 19.9) response rate, comorbidities, medications, biliary stent implantation, and adverse events (AEs).

Medical records were used to collect clinical data, and mortality data were derived from the Polish national database. Laboratory analyses were conducted by the Diagnostic Department of the PIM MSWiA. Counts of inflammatory cells for calculating the immune inflammation biomarker NLR were taken from laboratory results, which were performed immediately prior to treatment initiation. The response rate in terms of reduction in Ca 19.9 was evaluated before the beginning of treatment and then every 12 weeks until disease progression.

Treatment characteristics included concomitant therapies, treatment duration, the number of discontinued cycles, dose reductions, and interruptions. Dose modifications were based on the Summary of Product Characteristics.

### 2.2. Statistical Analyses

The primary objective was PFS, defined as the time from the start of SL treatment to disease progression or all-cause death. Secondary objectives included overall response rate (ORR), OS, defined as the time from the start of SL treatment to death from any cause, and toxicity. The analysis was conducted using IBM PQStat version 1.8.6 and SPSS Statistics v.20. Median OS (mOS) and median PFS (mPFS) were estimated using the Kaplan–Meier method with 95% confidence intervals. The Cox proportional-hazards model was used for a multivariate analysis to identify predictive factors for survival. A *p*-value of ≤0.05 was regarded as statistically significant. The quantitative variables NLR and CA 19.9 were transformed into binary variables with cutoff values of 3 and 34 U/mL, respectively. Response to treatment was measured according to the Response Evaluation Criteria in Solid Tumors (RECIST), version 1.1. Radiological assessments using CT of the chest, abdominal cavity, and pelvis were conducted every 12 weeks.

Safety was assessed in terms of AEs graded according to the Common Terminology Criteria for Adverse Events (CTCAE) version 5.0 [23].

### 2.3. Ethics Approval and Consent to Participate

The work described in this article has been carried out in accordance with the Code of Ethics of the World Medical Association (Declaration of Helsinki) on medical research involving human subjects, which are the ethical principles defined in the Farmington Consensus of 1997. The study was acknowledged by the Bioethics Committee of the National Medical Institute of the Ministry of the Interior and Administration (PIM MSWiA/KB/020524/2024).

## 3. Results

### 3.1. Case Selections

The analysis covered a cohort of 251 patients undergoing treatment for mPC with FL chemotherapy using GEM-NAB from February 2017 to January 2025. Among them, 108 (43%) patients received SL chemotherapy, comprising 64 women (59%) and 44 men (41%) following FL disease progression.

### 3.2. Patient Characteristics

The age range of the research sample was 37 to 84 years, with a median age of 66 years (mean 64.8, standard deviation SD = 8.8). In the female subgroup, the median age was 65 years (mean 68, standard deviation SD = 9.96), while in the male subgroup, it was, respectively, 63.5 years (mean 62.5, standard deviation SD = 8.6). Eighty-six percent of patients had a baseline ECOG score of 1.

Thirty-nine patients received FOLFOX 6; twenty-two received FOLFIRI; twenty-one received FOLFIRINOX; nineteen received NALIRI; and seven received cisplatin-gemcitabine (GEM + cisplatin).

In our cohort, the location of PC was distributed as follows: 65% (70) in the head, 25% (27) in the body, and 11% (10) in the tail. A total of 38 (35%) patients underwent Whipple pancreaticoduodenectomy, and 15 (14%) underwent distal pancreatectomy. Twenty-nine percent of the patients received adjuvant chemotherapy. Metastases were identified in 80 (74%) patients in the liver, 27 (25%) in the lungs, 20 (19%) in the lymph nodes, and 10 (9%) in the peritoneum. Lung-only metastases were observed in 12% of patients.

Patients received between 2 and 34 chemotherapy cycles in total. Demographic, clinical, and pathological features are reported in Table 1 and Table 2.

Complete response (CR) was not observed in any patient. Partial response (PR) was observed in 7 out of 108 patients who had imaging performed by the end of data collection. Disease progression (PD) was achieved for 65 patients. Disease stabilization (SD) was observed in 25 patients. The remaining 10 patients maintained their treatment. A total of 108 patients had a baseline CA19.9 measurement. Eight percent of patients had a decrease from baseline of less than 50%, while ten percent had a decrease of at least 50% (Table 3).

### 3.3. Survival Analysis

**Figure 2:**
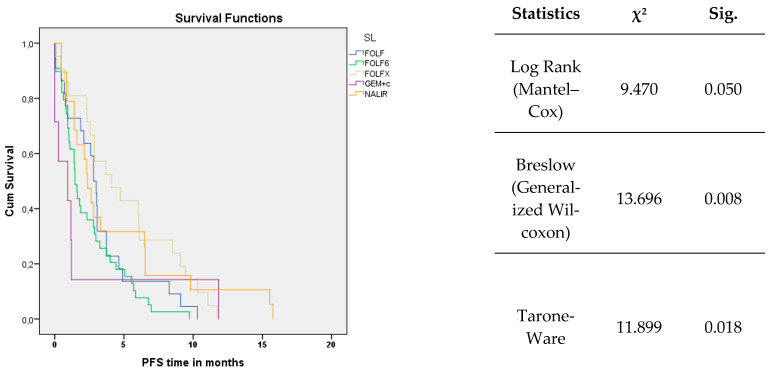
Kaplan–Meier curve for progression-free survival (PFS).

The mPFS in the observed group was 2.33 months (95% CI 1.65–3.01). For chemotherapy FOLFIRINOX, mPFS was 4.1 months (95% CI 1.31–6.90); for FOLFIRI, mPFS was 2.8 months (95% CI 2.30–3.30); for NALIRI, mPFS was 2.37 months (95% CI 1.66–3.08); for FOLFOX 6, mPFS was 1.47 months (95% CI 1.18–1.75); and for GEM-cisplatin, mPFS was 0.93 months (95% CI 0.00–2.64) (Figure 2).

The results of tests of equality of survival distributions for different levels were also obtained. Log Rank (Mantel–Cox) was χ^2^ = 9.470, *p*-val = 0.05, indicating that there were no statistical differences in the later treatment periods. Breslow (Generalized Wilcoxon) was χ^2^ = 13.696 with *p*-val = 0.008, indicating, at the α = 0.05 level of significance, significant differences in survival time during the initial treatment periods. Differences in mid-therapy periods were verified by the Tarone-Ware statistic and obtained at χ^2^ = 11.899 with *p*-val = 0.018, indicating significant differences in PFS for mid-therapy.

**Figure 3:**
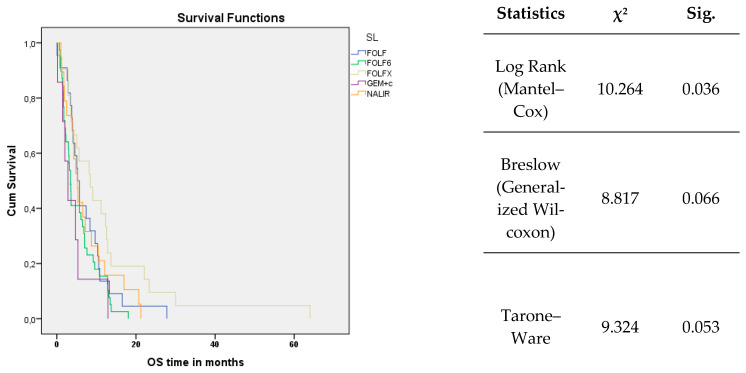
Kaplan–Meier curve for overall survival (OS).

The mOS in the observed group was 5.03 months (95% CI 3.76–6.31). The analysis did not reveal statistically significant differences in mOS based on the type of chemotherapy administered: for FOLFIRINOX, mOS was 8.5 months (95% CI 3.27–13.73); for FOLFIRI, mOS was 5.23 months (95% CI 3.93–6.53); for NALIRI, mOS was 5.23 months (95% CI 3.72–6.75); for FOLFOX 6, mOS was 3.47 months (95% CI 2.90–4.04); and for PG, mOS was 2.8 months (95% CI 0.83–4.77) (Figure 3).

The OS curves showed a statistically significant difference (log-rank test, χ^2^ = 10.264, *p* = 0.036). Visual inspection of the curves suggested that this difference became more pronounced in the later observation periods. The analysis of survival curves and associated statistics demonstrates that the therapies have statistically significant differential effects on OS and PFS. This outcome is crucial for informing treatment selection and optimizing therapeutic planning

The most common reasons for discontinuing treatment were PD (*n* = 66, 61%). After completion of treatment, 42 (39%) patients received a 3rd line of chemotherapy, a further 5 (5%) patients received a 4th line of chemotherapy, and 1 patient received a 5th line of chemotherapy.

The next point of analysis was the effectiveness of individual second-line therapy within the group. (Table 4).

In the study, physicians had six types of second-line therapies at their disposal. To verify the hypothesis of equality of the measure of central tendency and identity of distribution, the Kruskal–Wallis test and the median equality test for k independent samples were used. The obtained results are presented in Table 5.

For PFS, a test significance of *p* < 0.05 provides grounds for rejecting the null hypothesis in favor of the alternative hypothesis. This indicates significant differences in survival times and their distributions among groups after Category II SL therapy, necessitating the identification of the specific therapies for which these differences were observed (Table 6). The median overall PFS for patients was 2.37 months. The results indicate a statistically significant difference in PFS time between FOLFIRINOX and GEM + cisplatin therapies.

OS was assessed in a similar manner. The results are presented in Table 7.

In both tests analyzed, the *p*-value for OS was >0.05. This indicates that there is no reason to reject the null hypothesis of equality of medians and similarity of distributions according to the type of second-line therapy at the assumed level of significance. The mOS of patients was 5.03 months.

### 3.4. Safety

Treatments were well tolerated. AEs associated with treatment are presented in Table 8.

The most frequent of them included anemia, fatigue, peripheral neuropathy, neutropenia, and thrombocytopenia. The highest number of AEs occurred during treatment with FOLFIRINOX chemotherapy. Detailed information about the toxicity associated with individual SL treatment is available in Table 9.

The most common grade > 3 AEs were neutropenia and anemia (Table 10). Only 2% of patients experienced grade 4 AEs during FOLFIRINOX chemotherapy.

### 3.5. Predictive Factors

We analyzed the factors that significantly influenced OS and PFS for second-line therapy (Table 11).

Factors such as underweight (BMI < 18.5), neutropenia, use of 3rd and 4th line therapy, stable CA 19.9 antigen levels, a CA 19.9 decrease of less than 50%, and a CA 19.9 decrease of at least 50% impact both OS and PFS.

The alcohol consumption significantly influenced PFS but did not reach the significance threshold in the OS analysis. Similar relationships were identified for the following variables: overweight (BMI > 25), thrombocytopenia, and G-CSF use. These factors demonstrated a positive correlation with PFS but had an insignificant effect on OS. For NLR > 3 and Ca 19–9 > 34, an inverse relationship with PFS was observed, though only within one of the analyzed categories. Specifically, the presence of these factors demonstrated a negative direction of impact on PFS while having no significant effect on OS.

## 4. Discussion

Metastatic pancreatic cancer (mPC) is a highly aggressive malignancy that presents considerable challenges in its treatment. Alongside the ongoing debate about the optimal choice of FL treatment, it is becoming increasingly clear that SL therapies may play a significant role in the treatment algorithm for these patients. SL chemotherapy is an option for patients who maintain a good performance status after their disease has progressed on FL treatment. In the MPACT trial, 68% of patients, after FL therapy, had a Karnofsky performance status of 90–100 [24]. A total of 40% of patients in the MPACT trial received chemotherapy after progression on FL treatments. In our study, the majority of patients (86%) had an ECOG score of 1.

Currently, there is no standard SL treatment recommended for mPC. The treatment choice is based on many factors, such as the patient’s clinical condition, previous treatment, and pre-existing toxicities. After FL GEM-NAB treatment, multiple regimens including NALIRI, FOLFIRINOX, FOLFOX, FOLFIRI, or 5-FU-based monotherapy are listed in major guidelines as appropriate therapies [25].

In our study, among 251 patients who received FL therapy, 108 (43%) received SL treatment. These 108 patients were studied in six groups based on the treatment they received: FOLFOX 6, FOLFIRI, FOLFIRINOX, NALIRI, and GEM + cisplatin. The criteria for qualifying for specific second-line therapies included the age of the patients, the ECOG score, and the presence of peripheral polyneuropathy. Younger patients in good general condition (ECOG 1) were eligible for FOLFIRINOX chemotherapy. Patients who developed G2 or G3 peripheral polyneuropathy after FL therapy were eligible for treatments with NALIRI or FOLFIRI chemotherapy.

**Figure 4:**
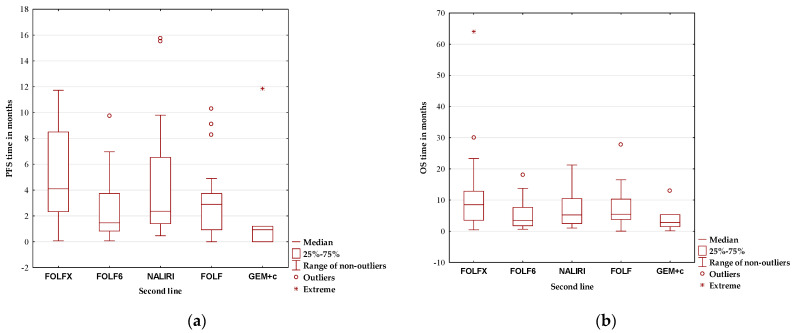
(**a**) Progression-free survival (PFS); (**b**) overall survival (OS).

The results indicate a statistically significant difference in PFS time between FOLFIRINOX and GEM + cisplatin therapies (Figure 4).

FOLFIRINOX chemotherapy showed the longest mPFS (4.1 months; 95% CI 1.31–6.90) and mOS (8.5 months; 95% CI 3.27–13.73). Many retrospective studies have evaluated the use of SL FOLFIRINOX in patients with disease progression following FL GEM-based treatments. A real-world Italian study compared SL FOLFIRINOX to FOLFOX 6 and FOLFIRI. Compared with FOLFIRI, FOLFIRINOX was reported to have significantly better median PFS and OS. However, no significant differences were observed between the FOLFIRINOX and FOLFOX groups in terms of PFS and OS [26].

Liposomal irinotecan is a molecule that can remain in the circulation longer than standard irinotecan and therefore can be more readily acquired by tumor cells [27]. Our analysis revealed no statistically significant difference between NALIRI and FOLFIRI. FOLFIRI chemotherapy showed an mPFS of 2.8 months (95% CI 2.30–3.30), while NALIRI mPFS was 2.37 months (95% CI 1.66–3.08). The mOS for both regimens was 5.23 months. In the NAPOLI-1 study, the median PFS was 3.1 months, and the median OS was 6.1 months [19]. Our study’s results are close to those. NALIRI was not superior to any other regimen in SL therapy. Therefore, when determining SL treatment for patients, costs should be taken into account.

FOLFIRI or NALIRI might be good treatment options for elderly patients [28]. The FOLFIRINOX option is reserved for highly selected patients who are mostly younger and fitter. The median age of patients receiving FOLFIRINOX in our study was 59 years.

Adverse events (AEs) are commonly observed in patients undergoing chemotherapy for mPC. Our study confirms these findings, indicating that FOLFIRINOX chemotherapy increases the risk of AEs. The most frequent of them included anemia, fatigue, peripheral neuropathy, neutropenia, and thrombocytopenia. It should be emphasized that, in patients who developed G1-G2 peripheral polyneuropathy following FL GEM-NAB chemotherapy, the condition did not significantly worsen during FOLFIRINOX therapy. No G3-G4 toxicity in terms of peripheral polyneuropathy has been reported in patients treated with SL FOLFIRINOX chemotherapy.

In addition to assessing the safety and effectiveness of SL therapy in real-life patients, we also analyzed prognostic factors and their influence on survival in this setting. Factors such as underweight (BMI < 18.5), neutropenia, use of 3rd and 4th therapy, stable CA 19.9 antigen levels, a CA 19.9 decrease of less than 50%, and a CA 19.9 decrease of at least 50% impact both OS and PFS. For NLR > 3 and Ca 19–9 > 34, an inverse relationship with PFS was observed, though only within one of the analyzed categories. Specifically, the presence of these factors reduced PFS while having no significant effect on OS.

We are conducting an increasing number of real-life studies on predictive biomarkers for specific FL therapies in mPC [29,30,31,32,33]. The influence of biomarkers such as TUBB3 and hENT1 on the response to GEM-NAB treatment is becoming an increasingly common topic of a lot of study. The absence of TUBB3 may be a favorable predictive marker of response to GEM-NAB in patients with mPC [34]. High expression of hENT1 mRNA strongly predicts a better response to GEM-NAB treatment and a longer OS in patients with mPC [35]. However, we still need to identify predictive biomarkers of response to SL treatment.

## 5. Conclusions

Our results showed that a fluoropyrimidine-containing treatment after FL may be an effective treatment sequence with favorable clinical benefits. The treatment is associated with an increased risk of manageable AEs. Further guidance on the use of SL therapy will depend on enhanced identification of biological predictors of SL treatment benefit. It will also require a better understanding of how effective FL therapy may influence subsequent treatment outcomes, giving indications on the importance of the analyzed factors for overall and disease progression-free survival.

## Figures and Tables

**Table 1:** Baseline demographic and clinical characteristics (*n* = 108).

Characteristics	Patients *n* (%)
No of patients	108 (100)
Average Median Age (range)	66 (37–84)
Sex	
Male	44 (41)
Female	64 (59)
ECOG score	
1	93 (86)
2	15 (14)
Site	
Head or neck	70 (65)
Body	27 (25)
Tail	11 (10)
Metastatic sites	
Liver	80 (74)
Lung	27 (25)
Lymph node	20 (19)
Peritoneal	10 (9)
Previous Whipple procedure	38 (35)
Previous distal pancreatectomy	15 (14)
Adjuvant chemotherapy, *n* (%)	31 (29)
mFOLFIRINOX	13 (12)
GEM	9 (8)
GEM + capecitabine	8 (7)
Hepatobiliary stent	21 (19)
Mechanical jaundice—1st symptom	23 (21)
BMI	
BMI < 18,5	6 (6)
BMI > 25	24 (22)
NLR, *n* (%)	
>3	56 (52)
<3	52 (48)
Ca 19.9, *n* (%)	
>34	89 (82)
<34	19 (18)
Common comorbidities, *n* (%)	
Hypertension	43 (40)
Diabetes	33 (31)
Thromboembolism	11 (10)
Autoimmune disease	8 (7)
Other cancer	7 (6)
Depression	6 (6)
2nd line, *n* (%)	108 (100)
FOLFOX 6	39 (36)
FOLFIRI	22 (20)
FOLFIRINOX	21 (19)
NALIRI	19 (18)
GEM + cisplatin	7 (6)
3rd line, *n* (%)	42 (39)
FOLFIRI	14 (13)
GEM + cisplatin	12 (11)
FOLFOX 6	9 (8)
NALIRI	2 (2)
FOLFIRINOX	2 (2)
GEM	2 (2)
Capecitabine	1 (1)
4th line	5 (5)
FOLFIRINOX	2 (2)
NALIRI	2 (2)
Docetaxel	1 (1)

Abbreviations: ECOG—Eastern Cooperative Oncology Group, BMI—Body Mass Index, NLR—Neutrophil to Lymphocyte Ratio, Ca 19.9—Carbohydrate Antigen 19–9, mFOLFIRINOX—(Modified) 5-Fluorouracil with Leucovorin, Irinotecan, and Oxaliplatin, GEM—Gemcitabine, FOLFOX 6—5-Fluorouracil with Leucovorin, Oxaliplatin, FOLFIRI—5-Fluorouracil with Leucovorin, Irinotecan, NALIRI—5-Fluorouracil with Leucovorin, Liposomal Irinotecan.

**Table 2:** Basic demographic and clinical characteristics of patients divided into specific types of second-line chemotherapy (*n* = 108).

Characteristics	FOLFOX 6	FOLFIRI	FOLFIRINOX	NALIRI	GEM + Cisplatin
Average Median Age (range)	69 (52–84)	66 (51–78)	59 (41–78)	63 (37–81)	59 (42–71)
Sex					
Male	14 (13)	9 (8)	8 (7)	10 (9)	3 (3)
Female	25 (23)	13 (12)	13 (12)	9 (8)	4 (4)
ECOG score					
1	35 (32)	22 (20)	21 (19)	19 (18)	7 (6)
2	4 (4)	0 (0)	0 (0)	0 (0)	0 (0)
Site					
Head or neck	29 (27)	13 (12)	13 (12)	13 (12)	4 (4)
Body	8 (7)	5 (5)	6 (6)	5 (5)	3 (3)
Tail	2 (2)	4 (4)	2 (2)	1 (1)	0 (0)
Metastatic sites					
Liver	29 (27)	7 (6)	16 (15)	17 (16)	4 (4)
Lung	12 (11)	17 (16)	3 (3)	7 (6)	1 (1)
Lymph node	5 (5)	5 (5)	2 (2)	5 (5)	1 (1)
Peritoneal	2 (2)	3 (3)	2 (2)	3 (3)	1 (1)
BMI					
BMI < 18.5	7 (6)	3 (3)	0 (0)	2 (2)	3 (3)
BMI > 25	12 (11)	8 (7)	10 (9)	8 (7)	0 (0)
Common comorbidities, *n* (%)					
Hypertension	17 (16)	4 (4)	7 (6)	4 (4)	1 (1)
Diabetes	11 (10)	6 (6)	6 (6)	6 (6)	1 (1)
Thromboembolism	4 (4)	0 (0)	4 (4)	3 (3)	0 (0)
Autoimmune disease	1 (1)	3 (3)	1 (1)	3 (3)	0 (0)
Other cancer	0 (0)	2 (2)	2 (2)	2 (2)	0 (0)
Depression	2 (2)	0 (0)	2 (2)	1 (1)	1 (1)

Abbreviations: ECOG—Eastern Cooperative Oncology Group, BMI—Body Mass Index, mFOLFIRINOX—(Modified) 5-Fluorouracil with Leucovorin, Irinotecan, and Oxaliplatin, GEM—Gemcitabine, FOLFOX 6—5-Fluorouracil with Leucovorin, Oxaliplatin, FOLFIRI—5-Fluorouracil with Leucovorin, Irinotecan, NALIRI—5-Fluorouracil with Leucovorin, Liposomal Irinotecan.

**Table 3:** Effectiveness of treatment and additional therapies (*n* = 108).

Parameters	Results *n* (%)
ORR, *n* (%)	7 (6)
CR, *n* (%)	0 (0)
PR, *n* (%)	7 (6)
SD, *n* (%)	25 (23)
PD, *n* (%)	66 (61)
Could not be evaluable, *n* (%)	10 (9)
Ca 19.9 response, *n* (%)	
Stable, *n* (%)	45 (42)
Increase, *n* (%)	43 (40)
Decrease < 50%, *n* (%)	9 (8)
Decrease > 50%, *n* (%)	11 (10)
Response to FL chemotherapy, *n*(%)	
CR, *n* (%)	0 (0)
PR, *n* (%)	21 (19)
SD, *n* (%)	58 (54)
PD, *n* (%)	29 (27)
Concomitant treatment, *n* (%)	
Darbepoetin alfa	14 (13)
LMWH	13 (12)
Statins	8 (7)

Abbreviations: ORR—overall response rate, CR—complete response, PR—partial response, SD—disease stabilization, PD—progression disease, Ca 19.9—carbohydrate antigen 19–9, LMWH—low molecular weight heparins, FL—first line.

**Table 4:** Descriptive statistics for different types of chemotherapy within second-line (SL) therapy.

SL Therapy	Statistics	Time OS	Time PFS
	Mean	7.39	3.27
FOLFIRI	Median	5.47	2.90
	IQR	6.68	3.07
	Mean	12.23	5.05
FOLFIRINOX	Median	8.50	4.10
	IQR	9.73	6.48
	Mean	5.42	2.44
FOLFOX 6	Median	3.47	1.47
	IQR	5.86	2.90
	Mean	5.42	2.44
NALIRI	Median	3.47	1.47
	IQR	5.86	2.90
	Mean	4.20	2.20
GEM + cisplatin	Median	2.80	0.93
	IQR	3.86	1.20

Abbreviations: SL—second line, OS—overall survival, PFS—progression-free survival response rate, GEM—gemcitabine, FOLFIRINOX—5-fluorouracil with leucovorin, irinotecan, and oxaliplatin, FOLFOX 6—5-fluorouracil with leucovorin, oxaliplatin, FOLFIRI—5-fluorouracil with leucovorin, irinotecan, NALIRI—5-fluorouracil with leucovorin, liposomal irinotecan.

**Table 5:** Kruskal–Wallis and median tests results for progression-free survival (PFS) across therapy type.

Hypothesis Test Summary			
Null Hypothesis	Test	Sig.	Decision
The medians of Time PFS (30) are the same across categories of SL	Independent-Sample Median Test	0.016	Reject the null hypothesis
The distribution of Time PFS (30) is the same across categories of SL	Independent-Samples Kruskal–Wallis Test	0.022	Reject the null hypothesis

Asymptotic significances are displayed. The significance level is 0.05.

**Table 6:** Detailed identification of differences in progression-free survival (PFS).

	FOLFIRINOX	FOLFOX6	NALIRI	FOLFIRI	GEM + Cisplatin
FOLFIRINOX		0.09	1.00	1.00	0.049
FOLFOX6	0.09		1.00	1.00	1.00
NALIRI	1.00	1.00		1.00	0.39
FOLFIRI	1.00	1.00	1.00		0.60
GEM + cisplatin	0.049	1.00	0.39	0.60	

*p*-values for multiple comparisons are adjusted with Statistica 13.3 built-in correction. Abbreviations: FOLFIRINOX—5-fluorouracil with leucovorin, irinotecan, and oxaliplatin, GEM—gemcitabine, FOLFOX 6—5-fluorouracil with leucovorin, oxaliplatin, FOLFIRI—5-fluorouracil with leucovorin, irinotecan, NALIRI—5-fluorouracil with leucovorin, liposomal irinotecan.

**Table 7:** Kruskal–Wallis and median test results for overall survival (OS) across therapy type.

Hypothesis Test Summary			
Null Hypothesis	Test	Sig.	Decision
The medians of Time OS (30) are the same across categories of SL	Independent-Samples Median Test	0.415	Retain the null hypothesis
The distribution of Time OS (30) is the same across categories of SL	Independent-Samples Kruskal–Wallis Test	0.077	Retain the null hypothesis

Asymptotic significances are displayed. The significance level is 0.05.

**Table 8:** Treatment-associated toxicity (*n* = 108).

AEs of Any Grade	No of Patients (%)
Hematologic	
Anemia	85 (79)
Neutropenia	38 (35)
Thrombocytopenia	37 (34)
Non-hematologic	
Fatigue	69 (64)
Peripheral neuropathy	40 (37)

Abbreviations: AEs-adverse events.

**Table 9:** Toxicity associated with individual second-line (SL) therapies.

Adverse Events of Any Grade	FOLFOX 6 No of Patients 39 (*n*%)	FOLFIRI No of Patients 22 (*n*%)	FOLFIRINOX No of Patients 21 (*n*%)	NALIRI No of Patients 19 (*n*%)	GEM + Cisplatin No of Patients 7 (*n*%)
Hematologic					
Anemia	29 (74)	18 (82)	18 (86)	14 (74)	6 (86)
Neutropenia	10 (26)	9 (41)	12 (57)	7 (37)	0 (0)
Thrombocytopenia	14 (36)	6 (27)	8 (38)	5 (26)	4 (57)
Non-hematologic					
Fatigue	25 (64)	13 (59)	13 (62)	12 (63)	2 (29)
Peripheral neuropathy	8 (21)	5 (23)	10 (48)	9 (47)	2 (29)

Abbreviations: FOLFIRINOX—5-fluorouracil with leucovorin, irinotecan, and oxaliplatin, GEM—gemcitabine, FOLFOX 6—5-fluorouracil with leucovorin, oxaliplatin, FOLFIRI—5-fluorouracil with leucovorin, irinotecan, NALIRI—5-fluorouracil with leucovorin, liposomal irinotecan.

**Table 10:** Adverse events graded according to Common Terminology Criteria for Adverse Events version 5.0 (*n* = 108).

Adverse Events of Any Grade	FOLFOX 6 No of Patients 39 (*n*%)	FOLFIRI No of Patients 22 (*n*%)	FOLFIRINOX No of Patients 21 (*n*%)	NALIRI No of Patients 19 (*n*%)	GEM + Cisplatin No of Patients 7 (*n*%)
Hematologic					
Anemia G1-G2	25 (64)	18 (82)	17 (81)	11 (58)	4 (57)
Anemia G3	4 (10)	0 (0)	1 (5)	3 (16)	2 (29)
Neutropenia G1-G2	5 (13)	8 (36)	8 (38)	6 (32)	0 (0)
Neutropenia G3	3 (8)	1 (5)	4 (19)	1 (5)	0 (0)
Neutropenia G4	2 (5)	0 (0)	0 (0)	0 (0)	0 (0)
Thrombocytopenia G1-G2	14 (36)	6 (27)	7 (33)	4 (21)	4 (57)
Thrombocytopenia G3	0 (0)	0 (0)	1 (5)	1 (5)	(0)
Non-hematologic					
Fatigue G1-G2	25 (64)	13 (59)	13 (62)	12 (63)	2 (29)
Peripheral neuropathy G1-G2	6 (15)	4 (18)	10 (48)	9 (47)	0 (0)
Peripheral neuropathy G3	2 (5)	1 (5)	0 (0)	0 (0)	2 (29)

Abbreviations: FOLFIRINOX—5-fluorouracil with leucovorin, irinotecan, and oxaliplatin, GEM—gemcitabine, FOLFOX 6—5-fluorouracil with leucovorin, oxaliplatin, FOLFIRI—5-fluorouracil with leucovorin, irinotecan, NALIRI—5-fluorouracil with leucovorin, liposomal irinotecan.

**Table 11:** Multivariate analysis for overall survival and progression-free survival.

Correlations	Time *OS*	Time *PFS*
Alcohol	0.173	0.201 *
BMI < 18.5	−0.231 *	−0.228 *
BMI > 25	0.172	0.206 *
NLR > 3	−0.108	−0.193 *
Ca 19.9 > 34	−0.135	−0.284 *
Decrease Ca 19.9 < 50%	0.221 *	0.242 *
Decrease Ca 19.9 > 50%	0.383 *	0.396
Stable Ca 19.9	−0.358 *	−0.269 *
Neutropenia	0.377 *	0.436 *
Thrombocytopenia	0.173	0.208 *
G-CSF	0.142	0.270 *
3rd line	0.536 *	0.363 *
4th line	0.283 *	0.197 *

* significance at α = 0.05. Abbreviations: BMI—body mass index, NLR—neutrophil to lymphocyte ratio, Ca 19.9—carbohydrate antigen 19–9, G-CSF—granulocyte colony-stimulating factor.

## Data Availability

The data presented in this study are available on request from the corresponding author.

## Abbreviations

The following abbreviations are used in this manuscript:

AEs	Adverse events
BMI	Body mass index
CA19–9	Carbohydrate antigen 19–9
CR	Complete response
CT	Computed tomography
CTCAE	Common terminology criteria for adverse events
ECOG	Eastern cooperative oncology group performance status
FL	First-line
GEM	Gemcitabine monotherapy
GEM-NAB	Gemcitabine, nab-paclitaxel
KRN	National cancer registry
LMWH	Low molecular weight heparins
MH	Minister of health
mOS	Median overall survival
mPC	Metastatic pancreatic cancer
mPFS	Median progression-free survival
NLR	Neutrophil to lymphocyte ratio
ORR	Overall response rate
OS	Overall survival
PC	Pancreatic cancer
PD	Progression disease
PFS	Progression-free survival
PR	Partial response
RECIST	Response evaluation criteria in solid tumors
SD	Disease stabilization
SL	Second-line
